# Association between blood pressure levels and risk of stroke in patients with atrial fibrillation: results from a prospective registry in Xinjiang, China

**DOI:** 10.3389/fcvm.2026.1759080

**Published:** 2026-05-29

**Authors:** Bizhong Che, Meidina Yeerken, Kangting Luo, Wen Peng, Meng Wei, Xu Yang, Yanmei Lu, Xianhui Zhou, Hua Wang, Fubing Ma, Yunguo Chen, Youfa Wang, Baopeng Tang

**Affiliations:** 1 Department of Endocrinology, The First Affiliated Hospital of Xi’an Jiaotong University, Xi’an, China; 2 Global Health Institute, School of Public Health, Xi’an Jiaotong University, Xi’an, China; 3 Department of Cardiac Pacing and Electrophysiology, The First Affiliated Hospital of Xinjiang Medical University, Urumqi, China; 4 Xinjiang Key Laboratory of Cardiac Electrophysiology and Remodeling, The First Affiliated Hospital of Xinjiang Medical University, Urumqi, China; 5 Department of Public Health, Qinghai University Medical College, Xining, China; 6 Qinghai Provincial Key Laboratory of Prevention and Control of Glucolipid Metabolic Diseases with Traditional Chinese Medicine, Medical College, Qinghai University, Xining, China; 7 The Fourth Division Hospital, Xinjiang Production and Construction Corps, Yining, China; 8 Public Health Center, The First Affiliated Hospital of Xi’an Jiaotong University, Xi’an, China

**Keywords:** atrial fibrillation, blood pressure, in-hospital, older adults, stroke

## Abstract

**Background:**

The relationship between blood pressure (BP) levels upon hospital admission and in-hospital stroke in patients hospitalized with atrial fibrillation (AF) remains unclear and has been complicated by current guidelines with two different thresholds (≥140/90 mm Hg and ≥130/80 mm Hg) for treating hypertension.

**Methods:**

We retrospectively analyzed consecutive patients hospitalized with AF at the First Affiliated Hospital of Xinjiang Medical University, China, between January 2010 and April 2025. The association between BP levels and the risk of stroke was analyzed by logistic regression model. Restricted cubic spline models were used to provide more precise estimates and to explore the shapes of the relationships of systolic BP (SBP) and diastolic BP (DBP) with stroke.

**Results:**

A total of 25 638 patients hospitalized with AF were included in this study. Compared with participants with SBP/DBP <120/80 mmHg, multivariable-adjusted odds ratios (95% CIs) of stroke were 1.27 (1.16–1.39), 1.26 (1.11–1.45), and 1.76 (1.44–2.15) for those with SBP/DBP 140–159/90–99 mmHg, 160–179/100–109 mmHg, and ≥180/110 mmHg, respectively. Restricted cubic spline regression models showed linear dose-response relationships of SBP and DBP with stroke. Sensitivity and subgroup analyses further confirmed these results.

**Conclusions:**

Elevated BP level was independently associated with a higher risk of stroke among patients with AF in Xinjiang, China. Recognizing and controlling elevated BP would be important to optimally reduce the risk of stroke.

## Highlights

- Elevated BP level was associated with a higher risk of stroke in patients with AF.
- SBP and DBP showed linear dose-response with stroke.
- Adding BP to conventional risk factors improved risk prediction for stroke.

## 1 Introduction

Atrial fibrillation (AF) is the most commonly sustained disorder of cardiac rhythm and is associated with an increased risk of stroke (1). According to the American Heart Association (AHA)/American Stroke Association, stroke is defined as a neurological deficit attributed to an acute focal injury of the central nervous system by a vascular cause, incorporating both clinical and tissue-based criteria. This updated definition is particularly relevant for AF, in which cardioembolic cerebral infarction represents one of the most serious and devastating complications (2).

Hypertension is the most common comorbidity in patients with AF and is prevalent in approximately 80%–90% of subjects with AF (3, 4). Diagnosed hypertension is an important risk factor for ischaemic stroke in patients with AF and is incorporated in the CHA_2_DS_2_-VASc stroke risk prediction scheme, which is widely used in most guidelines for stroke prevention in AF (5, 6). Previous studies have shown that a high blood pressure (BP) was associated with an increased risk of stroke. Those studies have either focused on systolic BP (SBP) (7, 8) or on outcomes following hospital discharge in patients with AF (9–11). During hospitalization, patients are more likely to receive comprehensive management, including rhythm or heart rate control and antihypertensive treatment, that effectively lowers BP. Accordingly, understanding the association between BP levels and in-hospital outcomes is essential to optimizing the acute management of AF during admission. Furthermore, optimal control of BP should be considered an essential component of treating AF and undertaken within a strategy of comprehensive risk factor management (12–14). The 2024 ESC/EACTS management of AF guidelines recommended that patients with AF should have an antihypertensive target of BP <130/80 mm Hg (15). There is additional controversy regarding the dose-response relationship between BP and stroke; some sources have shown a linear relationship between BP and stroke incidence (16, 17), which is particularly important because a lower BP target may be beneficial for patients with AF. However, several important gaps remain. First, epidemiological and clinical data focusing specifically on hospitalized Chinese patients with AF are still limited, especially from northwestern and multi-ethnic settings such as Xinjiang. Second, most prior studies have emphasized long-term outcomes after discharge, whereas evidence regarding in-hospital stroke risk according to admission BP level is scarce. Third, whether the association between BP and stroke during hospitalization is robust across different BP thresholds relevant to contemporary guideline targets has not been fully clarified (18).

This study elucidated the relationship between BP levels upon hospital admission and in-hospital stroke in patients hospitalized with AF from a large registry and examined whether the relationship was influenced by the choice of treatment target of hypertension. We aimed to establish an evidence-based foundation for refining in-hospital BP management strategies in patients with AF.

## 2 Methods

### 2.1 Patients and study design

From a prospective registry of patients diagnosed with AF at the First Affiliated Hospital of Xinjiang Medical University, we retrospectively analyzed consecutive patients between January 2010 and April 2025. Patients aged 18 years or older diagnosed with AF during a previous or current hospitalization, confirmed by electrocardiogram (ECG) findings indicating the absence of *P* waves replaced by irregular, varying F waves on a single-lead ECG (lasting ≥30 s) or a 12-lead ECG (lasting ≥10 s) with absolutely irregular RR intervals were included. All data were centrally collected and checked for completeness and consistency. The procedures followed were in accordance with institutional guidelines. The study protocol was approved by the Ethics Committee of the First Affiliated Hospital of Xinjiang Medical University (Xinjiang, China), and individual informed consent was waived.

### 2.2 Data collection

Data collection was performed by extracting medical records of patients hospitalized with AF, including demographic characteristics, clinical features, lifestyle factors, in-hospital treatments, medical history, and use of medications upon admission. The BP measurements were obtained at admission using a standard mercury sphygmomanometer according to a standard protocol adapted from procedures recommended by the American Heart Association.

### 2.3 Outcomes

The in-hospital outcome of this study was stroke. All stroke cases were diagnosed by physicians during the hospitalization of patients and were documented in medical records. The diagnosis of stroke relied on the criteria set by the AHA/American Stroke Association, which took into account both neurological signs and symptoms, as well as imaging tests such as computed tomography scans or magnetic resonance imaging (2).

### 2.4 Statistical analysis

Patients hospitalized with AF were divided into five groups according to the SBP/diastolic BP (DBP) level (mmHg) measured upon admission: <120/ <80, 120–139/80–89, 140–159/90–99, 160–179/100–109, and ≥180/110 according to current Chinese clinical guideline (19). If the levels of SBP and DBP were of different grades, then the higher grade was chosen. Patient characteristics were presented and compared among the five groups. Continuous variables were expressed as means with SD or median with interquartile range (IQR), as appropriate. Categorical variables were described by frequencies with percentages. The generalized linear regression analysis was used to test for trend across different BP levels for continuous variables, and the Cochran-Armitage trend *χ*2 test was applied for categorical variables. Binary logistic regression models were conducted to estimate the risk of stroke. We calculated the odds ratios and 95% CIs for the higher BP levels compared with the lowest BP levels. Tests for linear trends in odds ratios across BP levels were conducted using the median within each group as the predictor. Potential covariates for stroke were selected based on prior knowledge. We performed 3 logistic regression models. The covariates included in the multivariable models were selected based on the findings in the univariate analyses, clinical relevance, and prior literature (20). Model 1 was an unadjusted model. Model 2 adjusted for age, sex, ethnicity (Han, Uyghur, Kazakh, Hui, or other), education (junior high school or below, high school, or college or above), body mass index, current smoking, and alcohol drinking. Model 3 included the factors in model 2 and etiological diagnosis of AF, medication (anticoagulant, antiarrhythmic, antiplatelet/aspirin, and antihypertensive), medical history (hypertension, diabetes mellitus, dyslipidemia, coronary heart disease), catheter ablation, and the length of hospital stay. Restricted cubic spline models were used to provide more precise estimates and to explore the shapes of the relationships of SBP and DBP with stroke with 4 knots (at the 5th, 35th, 65th, and 95th percentiles).

Subgroup analysis was performed to test whether the association between BP levels and stroke was modified by age (<65, 65–74, or ≥75 years), sex, education, etiological diagnosis of AF, history of hypertension, history of coronary heart disease, in-hospital anticoagulation, and catheter ablation. The interaction between BP levels and covariates of interested on stroke was tested using the likelihood ratio test of models by adding interaction terms, adjusting for the covariates in the model 3. Sensitivity analysis was carried out by dividing patients into four groups according to BP level (mmHg): <130/80, 130–139/80–89, 140–159/90–99, and ≥160/100 (10) and calculating the odds ratios and 95% CIs for the higher BP levels compared with <130/80 mmHg. In an additional sensitivity analysis, we further examined the associations of BP levels upon admission with stroke subtypes, including ischemic stroke and hemorrhagic stroke, using separate logistic regression models with the same covariate adjustment strategy as in the primary analysis. Furthermore, we assessed the performance of model with BP to predict the risk of stroke in patients with AF. First, the C statistic was calculated to evaluate the incremental value of SBP and DBP over conventional risk factors for discrimination between patients with and without stroke. Second, integrated discrimination improvement (IDI) and the category-free net reclassification improvement (*N*RI) were calculated to evaluate improvement in risk classification by adding SBP and DBP to the basic model with conventional risk factors. Multiple imputation for missing data was performed using the Markov chain Monten Carlo method. All two-sided *P* values < 0.05 were considered to be statistically significant. Statistical analysis was conducted using the SAS statistical software, version 9.4 (SAS Institute Inc., Cary, NC).

## 3 Results

### 3.1 Participant characteristics

After excluding patients with missing records on the BP level upon admission, 25 638 patients were assessed in the present study. Participant characteristics both overall and stratified by BP levels were presented in Table 1. Of 25 638 included patients hospitalized with AF, 10 221 (39.9%) were women, 17 164 (67.0%) were Han, and the mean (SD) age at the time of enrollment was 66.5 (12.9) years. The median length of stay for hospitalized patients with AF was 7 (IQR 5–10) days. The proportion of patients with <120/ <80 mmHg, 120–139/80–89 mmHg, 140–159/90–99 mmHg, 160–179/100–109 mmHg, and ≥180/110 mmHg was 31.9% (*n* = 8,172), 40.4% (*n* = 10 367), 19.3% (*n* = 4,946), 6.3% (*n* = 1,617), and 2.1% (*n* = 536), respectively. Compared with patients with lower BP levels, those with higher BP were older, more likely to be Kazakh and nonvalvular AF, and had lower education level and higher body mass index. Furthermore, patients with higher BP levels had higher prevalence of hypertension, diabetes, and coronary heart disease, and had a longer hospital stay than those with lower BP levels.

**Table 1:** Characteristics of patients hospitalized with atrial fibrillation by blood pressure (BP) levels in xinjiang, China.

Characteristics^a^	Total (*N* = 25 638)	BP levels upon admission, mmHg					
		Normal BP <120/80 (*n* = 8,172)	Normal-High BP 120–139/80–89 (*n* = 10 367)	Grade 1 Hypertension 140–159/90–99 (*n* = 4,946)	Grade 2 Hypertension 160–179/100–109 (*n* = 1,617)	Grade 3 Hypertension ≥180/110 (*n* = 536)	*P*_trend_
Age, years	66.5 ± 12.9	64.1 ± 13.7	67.0 ± 12.7	68.5 ± 11.9	69.2 ± 11.5	68.8 ± 12.6	<0.001
Age group, years							
<65	10 376 (40.5)	3,924 (48.0)	4,019 (38.8)	1,722 (34.8)	521 (32.2)	190 (35.5)	<0.001
65–74	7,368 (28.7)	2,163 (26.5)	3,067 (29.6)	1,498 (30.3)	495 (30.6)	145 (27.0)	<0.001
≥75	7,894 (30.8)	2,085 (25.5)	3,281 (31.6)	1,726 (34.9)	601 (37.2)	201 (37.5)	<0.001
Female	10 221 (39.9)	3,311 (40.5)	4,057 (39.1)	1,999 (40.4)	637 (39.4)	217 (40.5)	0.68
Ethnicity							
Han	17 164 (67.0)	5,512 (67.4)	6,961 (67.1)	3,300 (66.7)	1,043 (64.5)	348 (64.9)	0.02
Uyghur	4,959 (19.3)	1,643 (20.1)	1,996 (19.3)	908 (18.4)	317 (19.6)	95 (17.7)	0.04
Kazakh	1,464 (5.7)	401 (4.9)	558 (5.4)	319 (6.4)	129 (8.0)	57 (10.6)	<0.001
Hui	1,277 (5.0)	370 (4.5)	540 (5.2)	258 (5.2)	87 (5.4)	22 (4.1)	0.16
Other	774 (3.0)	246 (3.0)	312 (3.0)	161 (3.3)	41 (2.5)	14 (2.6)	0.71
Education							
Junior high school or below	14 599 (56.9)	4,519 (55.3)	5,895 (56.9)	2,895 (58.5)	948 (58.6)	342 (63.8)	<0.001
High school	6,069 (23.7)	1,931 (23.6)	2,476 (23.9)	1,148 (23.2)	395 (24.4)	119 (22.2)	0.78
College or above	4,970 (19.4)	1,722 (21.1)	1,996 (19.2)	903 (18.3)	274 (16.9)	75 (14.0)	<0.001
Systolic BP, mmHg	125.0 ± 19.7	105.3 ± 9.6	124.9 ± 7.7	141.9 ± 10.2	157.7 ± 13.7	173.8 ± 20.0	<0.001
Diastolic BP, mmHg	76.0 ± 13.5	65.8 ± 8.2	76.2 ± 7.8	83.9 ± 10.4	91.6 ± 12.7	106.8 ± 35.7	<0.001
Body mass index, kg/m^2^	25.4 ± 4.2	25.1 ± 4.1	25.3 ± 4.2	25.8 ± 4.1	25.9 ± 4.3	25.6 ± 4.4	<0.001
Current smoker	6,979 (27.2)	2,324 (28.4)	2,781 (26.8)	1,268 (25.6)	445 (27.5)	161 (30.0)	0.08
Alcohol drinker	5,375 (21.0)	1,738 (21.3)	2,178 (21.0)	996 (20.1)	346 (21.4)	117 (21.8)	0.55
Total cholesterol, mg/dL	3.6 ± 1.0	3.6 ± 1.0	3.6 ± 1.0	3.6 ± 1.0	3.7 ± 1.0	3.6 ± 1.0	0.65
High-density lipoprotein cholesterol, mg/dL	1.0 ± 0.3	1.0 ± 0.3	1.0 ± 0.3	1.0 ± 0.3	1.0 ± 0.3	1.0 ± 0.3	0.88
Etiological diagnosis of atrial fibrillation							<0.001
Valvular	4,283 (16.7)	1,716 (21.0)	1,550 (15.0)	724 (14.6)	215 (13.3)	78 (14.6)	
Nonvalvular	21 355 (83.4)	6,456 (79.0)	8,817 (85.0)	4,222 (85.4)	1,402 (86.7)	458 (85.4)	
Medication							
Anticoagulant	14 348 (56.0)	4,608 (56.4)	5,755 (55.5)	2,774 (56.1)	913 (56.5)	298 (55.6)	0.79
Antiarrhythmic	17 263 (67.3)	5,549 (67.9)	6,958 (67.1)	3,294 (66.6)	1,109 (68.6)	353 (65.9)	0.36
Antiplatelet/aspirin	8,934 (34.8)	2,865 (35.1)	3,606 (34.8)	1,714 (34.6)	561 (34.7)	188 (35.1)	0.99
Antihypertensive	10 162 (39.6)	3,213 (39.3)	4,061 (39.2)	2,028 (41.0)	649 (40.1)	211 (39.4)	0.26
Medical history							
Hypertension	12 560 (49.0)	3,049 (37.3)	4,984 (48.1)	3,041 (61.5)	1,107 (68.5)	379 (70.7)	<0.001
Diabetes mellitus	4,708 (18.4)	1,265 (15.5)	1,872 (18.1)	1,062 (21.5)	386 (23.9)	123 (22.9)	<0.001
Dyslipidemia	626 (2.4)	189 (2.3)	257 (2.5)	117 (2.4)	42 (2.6)	21 (3.9)	0.14
Coronary heart disease	3,707 (14.5)	1,039 (12.7)	1,559 (15.0)	774 (15.6)	256 (15.8)	79 (14.7)	<0.001
Catheter ablation	2,149 (8.4)	667 (8.2)	879 (8.5)	433 (8.7)	135 (8.3)	35 (6.5)	0.90
Length of hospital stay, days	7 (5–10)	7 (5–10)	7 (5–10)	7 (5–10)	7 (5–11)	7 (5–12)	<0.001

a Continuous variables are expressed as mean ± standard deviation (SD) or as median (interquartile range). Categorical variables are expressed as frequency (percentage).

### 3.2 Associations between BP levels upon admission and in-hospital stroke

**Figure 1:**
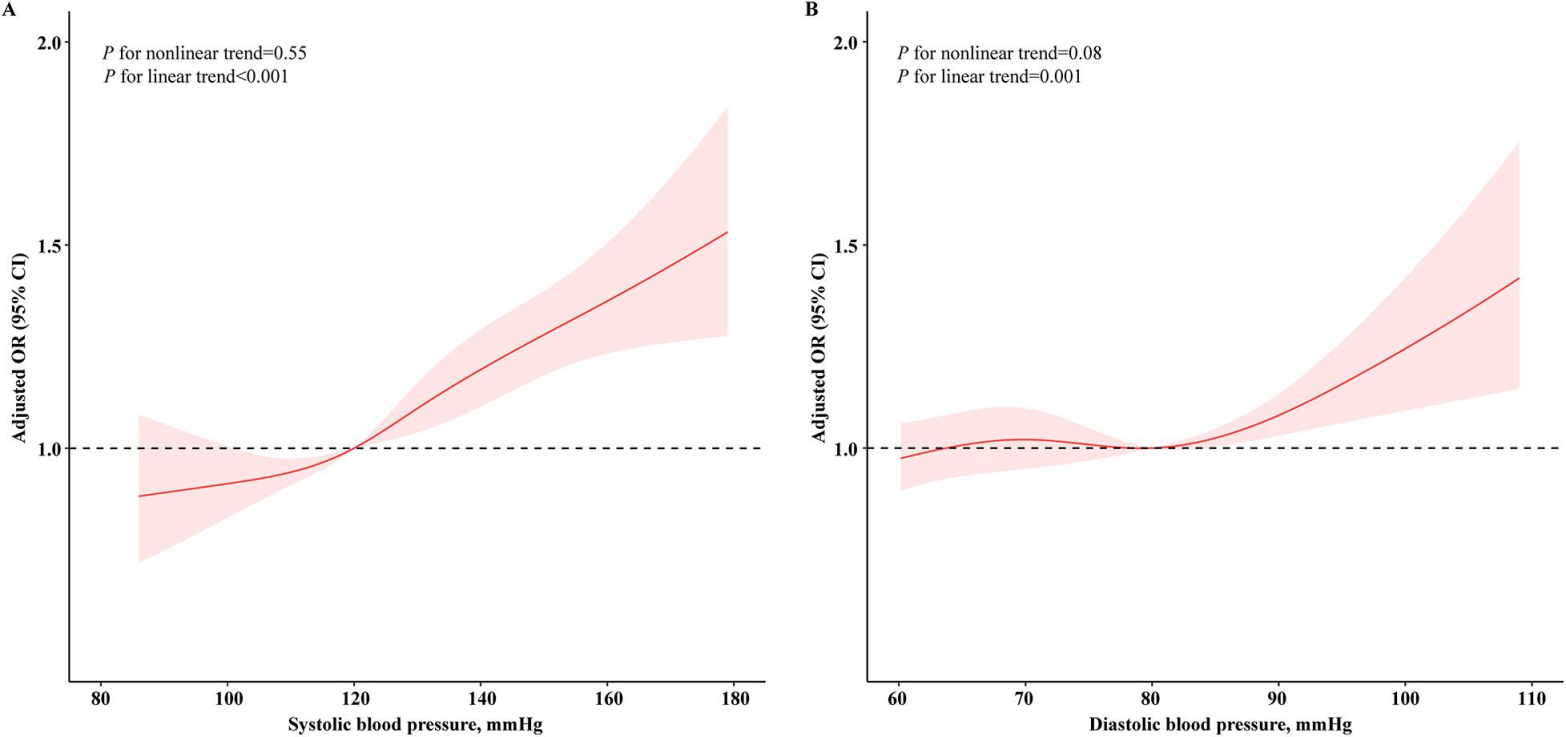
Association of systolic **(A)** and diastolic blood pressure **(B)** with risk of stroke. Odds ratios (OR) and 95% confidence intervals (CI) derived from restricted cubic spline regression, with knots placed at the 5th, 35th, 65th, and 95th percentiles of the distribution of blood pressure. The reference points for systolic and diastolic blood pressure are 120 mmHg and 80 mmHg, respectively. Odds ratios were adjusted for the same variables as model 3 in Table 2.

Among all patients hospitalized with AF, a total of 4,922 (19.2%) were defined as stroke. The rate of stroke was increased at higher BP levels, from 16.0% patients with BP <120/80 mmHg to 30.2% patients with BP ≥180/110 mmHg. In an unadjusted logistic regression model, BP level was positively associated with risk of stroke. This association remained significant after adjustment for age, sex, ethnicity, education, body mass index, current smoking, and alcohol drinking. After further adjustment for multiple potential confounders, including etiological diagnosis of atrial fibrillation, medical history (hypertension, diabetes mellitus, dyslipidemia, and coronary heart disease), catheter ablation, and the length of hospital stay, BP level was still significantly associated with an increased risk of stroke. Compared with participants with BP <120/80 mmHg, odds ratios (95% CIs) of stroke were 1.27 (1.16–1.39), 1.26 (1.11–1.45), and 1.76 (1.44–2.15) for those with BP 140–159/90–99 mmHg, 160–179/100–109 mmHg, and ≥180/110 mmHg, respectively (*P* trend < 0.001, Table 2). Multivariable adjusted spline regression models presented linear dose-response relationships of SBP (*P* for linearity <0.001; Figure 1A) and DBP (*P* for linearity=0.001; Figure 1B) with stroke.

**Table 2:** Odds ratios and 95% confidence intervals for the risk of stroke according to blood pressure levels .

	Blood pressure levels upon admission, mmHg					
	<120/80	120–139/80–89	140–159/90–99	160–179/100–109	≥180/110	*P* value for trend
Cases, *n* (%)	1,306 (16.0)	1,944 (18.8)	1,127 (22.8)	383 (23.7)	162 (30.2)	
Model 1	1.00	1.21 (1.12–1.31)	1.55 (1.42–1.70)	1.63 (1.43–1.86)	2.28 (1.88–2.76)	<0.001
Model 2	1.00	1.10 (1.02–1.19)	1.35 (1.23–1.47)	1.38 (1.21–1.58)	1.97 (1.62–2.40)	<0.001
Model 3	1.00	1.07 (0.99–1.16)	1.27 (1.16–1.39)	1.26 (1.11–1.45)	1.76 (1.44–2.15)	<0.001

Model 1: an unadjusted logistic regression.

Model 2: adjusted for age, sex, ethnicity, education level, body mass index, current smoking, and alcohol drinking.

Model 3: adjusted for model 2 and further adjusted for etiological diagnosis of atrial fibrillation, medication (anticoagulant, antiarrhythmic, antiplatelet/aspirin, and antihypertensive), medical history (hypertension, diabetes mellitus, dyslipidemia, and coronary heart disease), catheter ablation, and the length of hospital stay.

### 3.3 Subgroup and sensitivity analyses

Our subgroup analyses revealed that the aforementioned covariates (age, sex, education, etiological diagnosis of AF, history of hypertension, history of coronary heart disease, in-hospital anticoagulation, and catheter ablation) did not modify the relationship between BP and stroke (*P* > 0.1 for interaction for all, Table 3). Higher BP levels were significantly associated with stroke in most strata. In the sensitivity analysis, compared with participants with BP <130/80 mmHg, multivariable adjusted odds ratios (95% CIs) of stroke were 1.09 (1.01–1.18), 1.26 (1.16–1.37), and 1.37 (1.23–1.54) for those with BP 130–139/80–89 mmHg, 140–159/90–99 mmHg, and ≥160/100 mmHg, respectively (*P* trend <0.001, Table 4). Sensitivity analyses using ischemic stroke and hemorrhagic stroke as separate outcomes showed results generally consistent with those of the primary analysis, with higher BP levels upon admission being associated with a higher risk of both stroke subtypes (Table 5).

**Table 3:** Association between blood pressure and stroke upon admission and stroke in different subgroups of patients hospitalized with atrial fibrillation.

	Adjusted OR (95% CI) for stroke^a^					
	<120/80 mmHg	120–139/80–89 mmHg	140–159/90–99 mmHg	160–179/100–109 mmHg	≥180/110 mmHg	*P-*interaction
Age, years						0.88
<65	1.00	1.08 (0.93–1.25)	1.40 (1.17–1.67)	1.25 (0.95–1.65)	1.73 (1.17–2.56)	
65–74	1.00	1.00 (0.87–1.15)	1.10 (0.93–1.29)	1.09 (0.86–1.39)	1.86 (1.29–2.67)	
≥75	1.00	1.17 (1.03–1.33)	1.39 (1.20–1.61)	1.47 (1.20–1.80)	1.86 (1.37–2.52)	
Sex						0.67
Male	1.00	1.05 (0.95–1.17)	1.29 (1.15–1.46)	1.29 (1.09–1.53)	1.88 (1.45–2.44)	
Female	1.00	1.10 (0.97–1.25)	1.24 (1.08–1.44)	1.23 (1.00–1.52)	1.60 (1.17–2.19)	
Education						0.43
Junior high school or below	1.00	1.13 (1.02–1.25)	1.28 (1.13–1.44)	1.23 (1.04–1.46)	1.70 (1.32–2.18)	
High school	1.00	1.00 (0.84–1.17)	1.21 (1.00–1.47)	1.41 (1.08–1.84)	2.06 (1.35–3.14)	
College or above	1.00	0.99 (0.81–1.20)	1.32 (1.06–1.65)	1.18 (0.84–1.66)	1.68 (0.95–2.98)	
Etiological diagnosis of atrial fibrillation						0.27
Valvular	1.00	1.04 (0.86–1.26)	1.17 (0.92–1.47)	1.00 (0.69–1.45)	1.27 (0.72–2.22)	
Nonvalvular	1.00	1.09 (0.99–1.18)	1.29 (1.17–1.43)	1.32 (1.14–1.52)	1.86 (1.50–2.30)	
History of hypertension						0.14
Yes	1.00	1.07 (0.96–1.19)	1.17 (1.04–1.32)	1.18 (1.01–1.39)	1.67 (1.32–2.11)	
No	1.00	1.06 (0.95–1.19)	1.42 (1.23–1.65)	1.42 (1.12–1.81)	1.91 (1.30–2.79)	
History of coronary heart disease						0.25
Yes	1.00	1.28 (1.06–1.54)	1.34 (1.07–1.67)	1.38 (1.01–1.89)	2.34 (1.45–3.78)	
No	1.00	1.03 (0.94–1.12)	1.25 (1.13–1.39)	1.24 (1.07–1.44)	1.66 (1.33–2.07)	
In-hospital anticoagulation						0.72
Yes	1.00	1.09 (0.98–1.21)	1.27 (1.12–1.44)	1.17 (0.98–1.41)	1.74 (1.33–2.28)	
No	1.00	1.05 (0.93–1.18)	1.26 (1.10–1.45)	1.38 (1.14–1.69)	1.79 (1.33–2.42)	
Catheter ablation						0.34
Yes	1.00	1.02 (0.77–1.35)	1.03 (0.74–1.44)	1.20 (0.74–1.93)	3.03 (1.44–6.37)	
No	1.00	1.08 (0.99–1.17)	1.29 (1.17–1.42)	1.27 (1.10–1.46)	1.70 (1.38–2.09)	

a Odds ratios (OR) were calculated after adjustment for the same variables as model 3 in Table 2, except for the stratified variable.

**Table 4:** Odds ratios and 95% confidence intervals for the risk of stroke according to blood pressure levels: sensitivity analysis.

	Blood pressure levels upon admission, mmHg				
	<130/80	130–139/80–89	140–159/90–99	≥160/100	*P* value for trend
Cases, *n* (%)	1,924 (16.7)	1,326 (18.9)	1,127 (22.8)	545 (25.3)	
Model 1	1.00	1.16 (1.07–1.25)	1.47 (1.35–1.60)	1.69 (1.52–1.88)	<0.001
Model 2	1.00	1.12 (1.03–1.21)	1.33 (1.22–1.45)	1.50 (1.34–1.68)	<0.001
Model 3	1.00	1.09 (1.01–1.18)	1.26 (1.16–1.37)	1.37 (1.23–1.54)	<0.001

Model 1: an unadjusted logistic regression.

Model 2: adjusted for age, sex, ethnicity, education level, body mass index, current smoking, and alcohol drinking.

Model 3: adjusted for model 2 and further adjusted for etiological diagnosis of atrial fibrillation, medication (anticoagulant, antiarrhythmic, antiplatelet/aspirin, and antihypertensive), medical history (hypertension, diabetes mellitus, dyslipidemia, and coronary heart disease), catheter ablation, and the length of hospital stay.

**Table 5:** Odds ratios and 95% confidence intervals for the risk of ischemic stroke and hemorrhagic stroke according to blood pressure levels: sensitivity analysis.

	Blood pressure levels upon admission, mmHg					
	<120/80	120–139/80–89	140–159/90–99	160–179/100–109	≥180/110	*P* value for trend
Ischemic stroke						
Cases, *n* (%)	1,264 (15.5)	1,830 (17.7)	1,066 (21.6)	358 (22.1)	145 (27.1)	
Model 1	1.00	1.17 (1.08–1.27)	1.50 (1.37–1.64)	1.55 (1.36–1.77)	2.03 (1.66–2.48)	<0.001
Model 2	1.00	1.06 (0.98–1.15)	1.30 (1.18–1.43)	1.31 (1.15–1.50)	1.74 (1.42–2.14)	<0.001
Model 3	1.00	1.04 (0.96–1.12)	1.23 (1.12–1.35)	1.22 (1.06–1.39)	1.58 (1.28–1.94)	<0.001
Hemorrhagic stroke						
Cases, *n* (%)	16 (0.2)	55 (0.5)	26 (0.5)	11 (0.7)	7 (1.3)	
Model 1	1.00	2.72 (1.56–4.75)	2.69 (1.44–5.03)	3.49 (1.62–7.54)	6.75 (2.77–16.47)	<0.001
Model 2	1.00	2.71 (1.55–4.75)	2.73 (1.46–5.12)	3.54 (1.63–7.69)	6.87 (2.80–16.84)	<0.001
Model 3	1.00	2.58 (1.45–4.57)	2.30 (1.21–4.37)	2.78 (1.26–6.11)	5.35 (2.15–13.30)	<0.001

Model 1: an unadjusted logistic regression.

Model 2: adjusted for age, sex, ethnicity, education level, body mass index, current smoking, and alcohol drinking.

Model 3: adjusted for model 2 and further adjusted for etiological diagnosis of atrial fibrillation, medication (anticoagulant, antiarrhythmic, antiplatelet/aspirin, and antihypertensive), medical history (hypertension, diabetes mellitus, dyslipidemia, and coronary heart disease), catheter ablation, and the length of hospital stay.

### 3.4 Incremental predictive value of BP level

Table 6 presented the performance of models with BP levels to predict stroke. Adding SBP and DBP to the basic model with conventional risk factors significantly improved discrimination for stroke, shown by the C statistics increasing from 0.657 to 0.661 (*P* for difference <0.001). The addition of SBP and DBP to the basic model significantly enhanced risk reclassification for stroke [category-free NRI, 10.1% [95% CI, 7.0%–13.2%]; *P* < 0.001; IDI, 0.3% [95% CI, 0.2%–0.4%]; *P* < 0.001].

**Table 6:** Discrimination and reclassification statistics for the risk of stroke by blood pressure.

Variable	C statistics		NRI, category free		IDI	
	Estimate (95% CI)	*P* value	Estimate (95% CI), %	*P* value	Estimate (95% CI), %	*P* value
Basic model	0.657 (0.651–0.663)		Reference		Reference	
Basic model + blood pressure	0.661 (0.656–0.667)	<0.001	10.1 (7.0–13.2)	<0.001	0.3 (0.2–0.4)	<0.001

NRI, net reclassification improvement; IDI, integrated discrimination index; CI, confidence interval.

Basic model included age, sex, ethnicity, education level, body mass index, current smoking, alcohol drinking, etiological diagnosis of atrial fibrillation, medication (anticoagulant, antiarrhythmic, antiplatelet/aspirin, and antihypertensive), medical history (hypertension, diabetes mellitus, dyslipidemia, and coronary heart disease), catheter ablation, and the length of hospital stay.

## 4 Discussion

In this retrospective study of over 25 000 patients with AF, we examined the relationship between different BP levels upon admission and the risk of stroke during hospitalization. We found that higher BP level upon admission was associated with an increased risk of stroke, independently of several established covariables, including age, education status, medication, medical history, and etiological diagnosis of AF. The findings were consistent across different subgroups and were not affected by the definition of hypertension (≥140/90 mm Hg or ≥130/80 mm Hg), and remained similar when ischemic stroke and hemorrhagic stroke were analyzed separately. Furthermore, adding BP levels to conventional risk factors improved risk prediction for stroke. Taken together, these findings suggest that admission BP is not merely a descriptive vital sign in hospitalized patients with AF, but a clinically meaningful marker that may help identify patients at increased short-term cerebrovascular risk.

Patients with hypertension were at higher risk of stroke compared with patients without hypertension, and higher BP values in patients with AF were associated with an increased risk of long-term adverse outcomes including stroke (7, 9–11, 21). Data on the association of BP level with in-hospital stroke in patients hospitalized with AF are scarce in China. The Improving Care for Cardiovascular Disease in China-AF project suggested that higher BP levels (SBP/DBP ≥160/100 and SBP ≥160 mmHg) upon admission were associated with an increased risk of stroke during hospitalization (18). Our results corroborated previous studies and extended their findings into multi-ethnic patients with AF in China. In the present study, we observed linear associations of SBP and DBP with the risk of stroke, with a 76% risk increase in stroke for patients with BP ≥180/110 mmHg compared with those with normal BP (<120/80 mmHg) based on current Chinese clinical guideline for preventing hypertension. Another important finding is that for patients included in the present study, 36% of patients who had a history of hypertension had elevated BP upon admission (SBP≥140 mm Hg and/or DBP ≥90 mm Hg). This highlights an opportunity to focus on BP management in those patients with AF, including those with a previous diagnosis of hypertension. Our findings are also directionally consistent with emerging recent evidence showing that elevated BP remains relevant to stroke risk across different AF populations, including younger adults, suggesting that the prognostic value of BP may extend across age strata and clinical contexts (18).

The linear relationship between BP and stroke suggested that current guideline-defined hypertension brackets may not capture the full extent of the relationship between BP and stroke in patients with AF. It was also indicative that progressively lower BP lower stroke risk in these patients, which corroborated previous findings in a network meta-analysis of the relationship between SBP and stroke risk among hypertensive subjects (22). The linear relationship appeared to be particularly important in the context of the lower BP targets that were included in the current guidelines in patients with AF (15, 23). The linear relationship was explained in part by the relationship to age and other covariates. We found that the relationship between BP and stroke was not altered by choice of threshold (≥140/90 mm Hg vs. ≥130/80 mm Hg) — further supporting guideline changes that tightened BP targets for patients with AF. From a clinical perspective, this observation argues against treating BP thresholds in hospitalized AF patients as purely dichotomous categories. Rather, BP may behave as a continuous risk marker, and even moderately elevated values may carry prognostic relevance when integrated with the broader thromboembolic profile of the patient (15).

Several potential pathophysiologic pathways have been proposed for linking high BP to an increased stroke risk. Hypertension could lead to left ventricular hypertrophy, left atrial enlargement/fibrosis, and diastolic dysfunction, all of which may contribute to the increased burden of AF and consequently increased risk of stroke (24–26). In addition, high BP increases the hemodynamic burden on the aorta, reducing arterial compliance and atherosclerosis occurrence (27). Altered arterial wall elasticity and function directly induce multiple atherosclerosis complications including stroke (28, 29). Beyond these structural mechanisms, elevated BP may promote stroke through several interrelated biological pathways. Chronic pressure overload contributes to endothelial dysfunction, impaired nitric oxide bioavailability, oxidative stress, and low-grade vascular inflammation, which together accelerate arterial stiffness and promote a prothrombotic state (30, 31). In patients with AF, hypertension-related atrial remodeling may enhance atrial myopathy, blood stasis, and thrombogenesis, thereby increasing the likelihood of embolic events (32–34). Elevated BP can also aggravate cerebral small-vessel vulnerability and impair cerebrovascular autoregulation, potentially lowering the threshold for ischemic injury during episodes of embolic or hypoperfusion stress (35).

The present findings have several practical implications. First, admission BP may serve as a simple and available marker for short-term stroke risk stratification in hospitalized patients with AF. Second, the observed incremental value of SBP and DBP for discrimination and reclassification suggests that BP information may complement, rather than duplicate, established clinical risk factors. Third, our results support the concept that AF management during hospitalization should not be restricted to rhythm control, rate control, and anticoagulation alone, but should also include early recognition and appropriate control of elevated BP as part of integrated care. This is particularly relevant in resource-variable settings, where easily obtainable parameters may have substantial utility for bedside decision-making.

The primary strengths of this study include the large sample size, multi-ethnic nature of the population, and standardized protocol and rigid quality control procedures for data collection and outcome assessment. These strengths enabled us to control important confounding factors in the multivariable models and to provide a more valid assessment of the association between BP level and the risk of stroke. Our results remained robust in subgroup and sensitivity analyses. In addition, the study specifically addresses an underexplored but clinically important period—the in-hospital phase—during which BP levels, treatment intensity, and acute cerebrovascular risk may interact in ways that are not captured by outpatient or post-discharge studies.

However, there were several limitations of our study. First, this was a single-center retrospective analysis based on a tertiary hospital registry, which may limit the generalizability of our findings to other healthcare settings and may also introduce referral bias related to case severity. External validation in other populations is therefore warranted. Second, BP classification was based on a single measurement obtained at admission, which may not fully capture short-term BP variability, dynamic BP changes during hospitalization, or the intensity and timing of antihypertensive treatment after admission. These factors may also influence in-hospital stroke risk. Third, despite adjustment for multiple confounders, we cannot exclude residual confounding by unmeasured or unknown confounders. Fourth, our data set did not include long-term outcomes after hospital discharge. Follow-up data and the long-term outcomes of these study patients will be assessed in the future. Finally, the potential effects of reverse causality cannot be excluded.

## 5 Conclusion

Elevated BP level was independently associated with a higher risk of stroke among patients with AF in Xinjiang, China. Recognizing and controlling elevated BP would be important to optimally reduce the risk of stroke.

## Data Availability

The raw data supporting the conclusions of this article will be made available by the authors, without undue reservation.
